# Randomized controlled trial of remote ischemic preconditioning in children having cardiac surgery

**DOI:** 10.1186/s13019-023-02450-8

**Published:** 2024-01-03

**Authors:** Yuk M. Law, Christine Hsu, Sangeeta R. Hingorani, Michael Richards, David M. McMullan, Howard Jefferies, Jonathan Himmelfarb, Ronit Katz

**Affiliations:** 1 Pediatric Cardiology, Department of Pediatrics, Seattle Children’s Hospital, 4800 Sand Point Way NE, Seattle, WA 98105 USA; 2 University of Washington School of Medicine, Seattle, WA 98115 USA; 3 Kaiser Permanente of Washington, Seattle, WA 98105 USA

**Keywords:** Congenital heart disease, Cardiothoracic surgery, Children, Remote ischemic preconditioning, Kidney injury

## Abstract

**Background:**

Children undergoing cardiac surgery are at risk for acute kidney injury (AKI) and cardiac dysfunction. Opportunity exists in protecting end organ function with remote ischemic preconditioning. We hypothesize this intervention lessens kidney and myocardial injury.

**Methods:**

We conducted a randomized, double blind, placebo controlled trial of remote ischemic preconditioning in children undergoing cardiac surgery. Pre-specified end points are change in creatinine, estimated glomerular filtration rate, development of AKI, B-type natriuretic peptide and troponin I at 6, 12, 24, 48, 72 h post separation from bypass.

**Results:**

There were 45 in the treatment and 39 patients in the control group, median age of 3.5 and 3.8 years, respectively. There were no differences between groups in creatinine, cystatin C, eGFR at each time point. There was a trend for a larger rate of decrease, especially for cystatin C (*p* = 0.042) in the treatment group but the magnitude was small. AKI was observed in 21 (54%) of control and 16 (36%) of treatment group (*p* = 0.094). Adjusting for baseline creatinine, the odds ratio for AKI in treatment versus control was 0.31 (*p* = 0.037); adjusting for clinical characteristics, the odds ratio was 0.34 (*p* = 0.056). There were no differences in natriuretic peptide or troponin levels between groups. All secondary end points of clinical outcomes were not different.

**Conclusions:**

There is suggestion of RIPC delivering some kidney protection in an at-risk pediatric population. Larger, higher risk population studies will be required to determine its efficacy.

*Trial registration and date*: Clinicaltrials.gov NCT01260259; 2021.

**Supplementary Information:**

The online version contains supplementary material available at 10.1186/s13019-023-02450-8.

## Introduction

Children undergoing cardiovascular surgery requiring cardiopulmonary bypass are at risk for end organ dysfunction post-operatively [1, 2]. This complication is particularly relevant in pediatric cardiac surgery as more infants are undergoing more complex procedures.

Earlier work showed promise in protection from ischemic reperfusion injury to the heart by pre-treating the myocardium with direct subclinical ischemia through coronary clamping [3]. The difficulty in applying this approach to human subjects led to studies targeting delivery of subclinical ischemia to a non-vital organ in a remote site, so-called remote ischemic preconditioning (RIPC). The exact mechanism is not well defined, but it is felt to be related to ameliorating the effects of reperfusion injury such as occurs with cardiopulmonary bypass. The preconditioning with reversible ischemia triggers cellular and molecular events to better adapt to the subsequent more potent reperfusion from ischemia (Johansen 2007). It is noteworthy that the preconditioning can be remote from the tissue or organ of interest, suggesting that the events or mediators following the reversible ischemic preconditioning is systemic. Subsequent studies also produced beneficial biological and clinical effects in non-primate animals and human subjects [3, 4]. Consequently, an at-risk population of children undergoing cardiac surgery were studied. Several groups have demonstrated a biological effect and a suggestion for clinical improvement in a low risk group undergoing low-complexity cardiac surgery [5–7]. Additional studies with a larger population undergoing more complex surgery did not produce clinical benefits with RIPC [8, 9]. In the Seattle Cardiorenal Ischemic Preconditioning Trial (SCRIPT), we targeted a population of children undergoing higher complexity procedures to further delineate the clinical and biological effects of RIPC. We specifically targeted the ability of RIPC to protect the kidneys. Our hypothesis is that compared to placebo, RIPC can lessen the injury to the myocardium and kidneys in children undergoing cardiac surgery.

## Patients and methods

### Study design

This was a randomized, double-blind, placebo controlled trial enrolling eligible patients in the pediatric age group undergoing predefined surgical procedures requiring cardiopulmonary bypass. Randomization was stratified by age, above versus below 30 days, and cardiovascular diagnosis/surgical complexity using the Risk Adjusted Classification for Congenital Heart Surgery (RACHS-1) score 2 vs. 3 vs. 4–6^10^ categories. The randomization sequence was generated by a biostatistician at Seattle Children’s Hospital. Sealed envelopes are placed in that sequence, by the stratum assigned, 1–6 from the 2 by 3 randomization of age versus RACHS-1 category, respectively. A preliminary analysis of the last 12 months’ cases was performed to assess our institution’s cardiopulmonary bypass and aortic cross clamp times in relation to cardiovascular diagnosis and RACHS-1 categories. This information provided assurance that the bypass and cross clamp times increase with RACHS-1 categories, and that there were a reasonable number of candidate cases to be stratified. The subjects, clinicians providing post-operative care, investigators, research team members (other than the personnel delivering the RIPC/sham treatment), anesthesiologists and surgeons present during the operation were blinded to the treatment delivered. This was an intention to treat analysis. The study was registered under Clinicaltrials.gov NCT01260259. Local institutional review board approval was obtained.

### Study population

Patients < 18 years of age at time of surgery with qualifying congenital heart defect (Additional file 1: Table S1) and procedure planned at Seattle Children’s Hospital were eligible for the study. In brief, the surgical procedure is such that cardiopulmonary bypass is considered standard for that defect and has a complexity category of 2 or greater under the RACHS-1 classification [10]. Exclusion criteria included the following during the preoperative period: contraindication to compression of lower extremity; body weight < 2 kg; active infection at time of surgery; on continuous renal replacement therapy, mechanical circulatory support; gestational age < 36 weeks; status as foster care or ward of the state.

### Remote ischemic preconditioning

Investigators (CH and YML) screened, consented, and assigned the stratum in the randomization. Research associates not involved in the data collection performed RIPC or sham intervention in the operating room.

After induction of anesthesia but before incision of the chest, intervention is delivered by placing a blood pressure cuff on a lower extremity. For the placebo group, the cuff stayed on uninflated for 40’. For the RIPC group, it was inflated to 15 mmHg above the systolic blood pressure as obtained by the anesthesiologist for 5’, deflated for 5’, and repeated for a total of 4 cycles totaling 40’. The treatment was planned and performed such that it did not interfere with the conduct of the operating room.

### Study bio-assays and end points

The primary end points are designed to demonstrate a kidney or cardioprotective effect from RIPC compared to placebo. The kidney protective effect is measured by the difference in the change in serum creatinine between groups. The cardioprotective effect is measured by the difference in the change in plasma troponin I as a marker of myocardial injury between groups. Blood samples for the measurement of creatinine and troponin I were collected preoperatively (0–7 days before procedure), and at 6, 12, 24, 48, 72 h after separation from cardiopulmonary bypass. The blood specimens were immediately transported to laboratory, centrifuged, and the serum/plasma frozen to − 80 °C. The serum creatinine and cystatin C levels were measured with a clinical chemistry analyzer (DXC600, Beckman Coulter, Miami, Florida). Creatinine levels were determined by the modified Jaffe method and cystatin C via a particle turbidimetric immunoassay (Gentian USA, Inc.). Testing for plasma troponin I was performed using the Alere Triage® kit (Alere, Waltham, MA).

Secondary kidney and myocardial protection end points included the change in cystatin C, estimated glomerular filtration rate (eGFR) calculated using serum creatinine (0.413 × (height/serum creatinine)) [11], eGFR calculated using cystatin C (70.69 × (cystatin C)^−0.931^) [12], and change in plasma B-type natriuretic peptide (BNP). Secondary clinical end points included AKI using the Kidney Disease: Improving Global Outcomes criteria of an increase in creatinine ≥ 0.3 mg/dL within 48 h or an increase ≥ 1.5 times baseline within 7 days [13], need for dialysis, new or worsened systemic ventricular systolic dysfunction on intraoperative transesophageal echocardiogram or on post-operative transthoracic echocardiogram before discharge.

### Statistical analysis

Baseline characteristics by treatment group were summarized using means, medians, or frequencies, as appropriate. Mean changes from baseline to designated time points within randomized treatment groups were analyzed as intent to treat using linear mixed models. In a secondary analysis, a logistic regression model was fitted with AKI as the outcome. We obtained odds ratios and 95% confidence intervals from this model and adjusted for baseline creatinine, age, sex, and race. Statistical analyses were performed with SPSS version 26.0 (IBM Corp.) and R version 4.0.2 (https://www.R-project.org/). A p-value of less than 0.05 was considered statistical significance, with no adjustments for multiple testing.

There was not robust data regarding the impact of RIPC on kidney function in children after cardiac surgery. Our power calculation was based on literature available on AKI in children after cardiac surgery [14]. For a power of 90% with a 5% chance of a Type I (alpha) error, we estimated that 100 patients were needed, randomized equally to treatment and control. This was powered to detect a 25% change in creatinine in the RIPC group compared to controls. If recruitment is < 100 patients, we will accept a study enrollment of 70 patients, yielding 80% power to detect a 25% change in creatinine between groups, with a 5% chance of a Type I error.

## Results

**Fig. 1:**
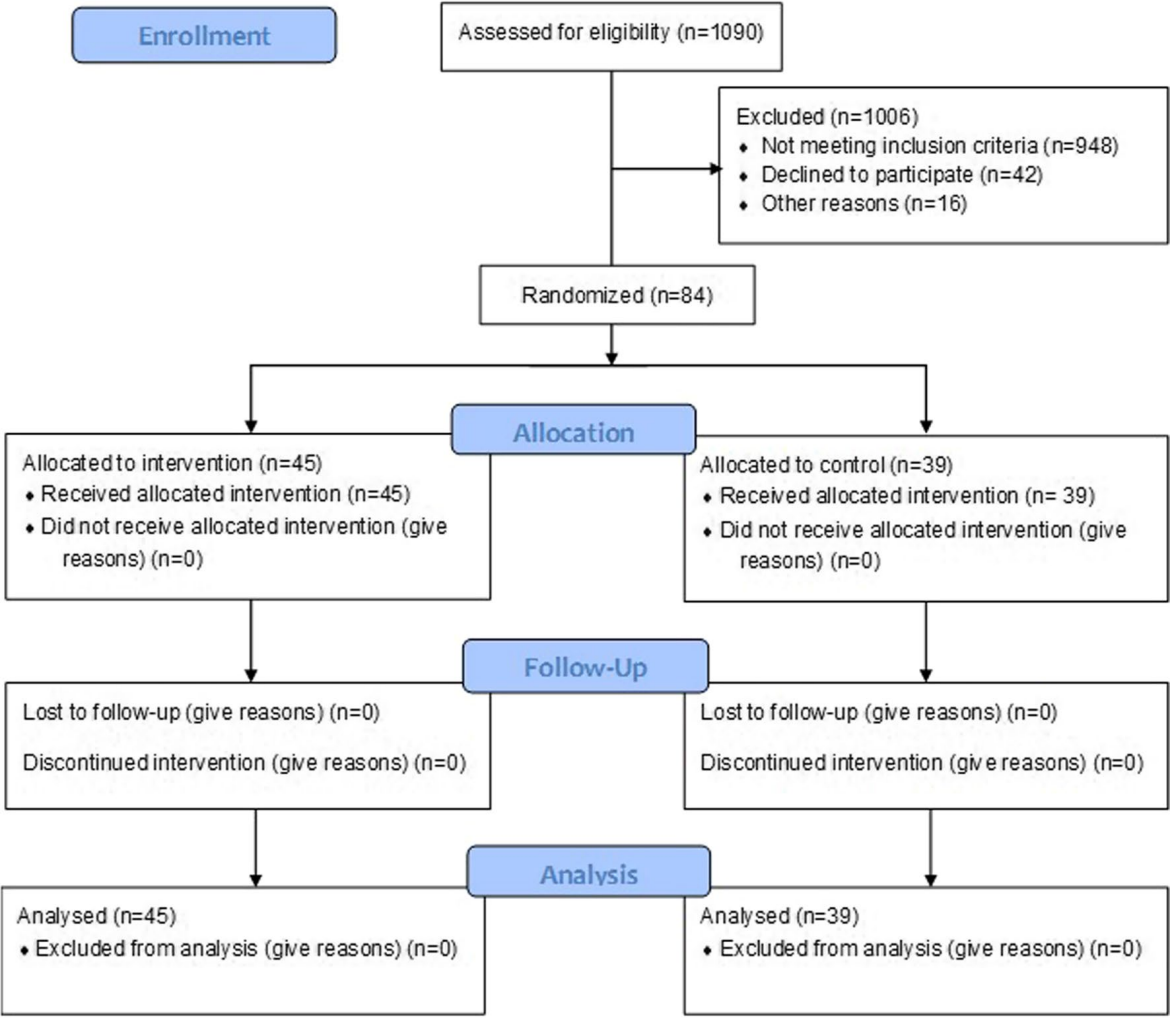
Consort diagram for the trial

From January 2011 to August 2013, a total of 100 patients were enrolled and 84 randomized: 39 in the control and 45 in RIPC arm. There were 16 patients not included in the study after consenting and before randomization because either the surgical procedure changed, or the timing did not allow for the intervention in the operating room (Fig. 1). The clinical characteristics of the groups are well balanced with most of the patients being Caucasian, not on cardiac medications, and of young age (Table 1). Although the cohort included older children, the median age was 0.38 to 0.42 years for the two groups, and hence many were infants with 57 below the age of 12 months, and 35 below the age of 1 month at the time of surgical intervention. This skewing to the side of infants in the study population is reflected in the type of the surgical procedure (Table 2). The higher risk procedures under RACHS-1 4–6 categories are typically undertaken in young infants with complex, hemodynamically significant, congenital heart disease. This risk category along with other aspects of the surgical procedure are well balanced between the two groups.

**Table 1:** Baseline clinical characteristics of patients

Clinical variables	Control	RIPC	Total
	(n = 39)	(N = 45)	(n = 84)
Median age at surgery (years with IQR)	0.38 [0.02, 6.05]	0.42 [0.02, 5.62]	0.36 [0.02, 5.62]
Male	24 (62%)	22 (49%)	46 (55%)
Weight in OR (kg)	5.7 [3.5, 18.6]	5.3 [3.4, 16.4]	5.4 [3.5, 17.8]
BSA in OR (m^2^)	0.34 [0.24, 0.79]	0.32 [0.23, 0.70]	0.32 [0.24, 0.74]
Presence of a clinical syndromic	5 (13%)	3 (7%)	8 (10%)
White race	25 (64%)	30 (67%)	55 (66%)
Preoperative diuretic	3 (8%)	8 (18%)	11 (13%)
Preoperative ACE-I or MRA	4 (10%)	3 (7%)	7 (8%)
Preoperative PGE	5 (13%)	6 (13%)	11 (13%)
Congenital heart disease diagnosis			
Single-ventricle	7 (18%)	9 (20%)	16 (18%)
Two-ventricle	32 (82%)	36 (80%)	68 (81%)
Preoperative creatinine (mg/dL)	0.36 [0.24, 0.50]	0.37 [0.22, 0.45]	0.37 [0.23, 0.48]
Preoperative troponin (ng/mL)	0.04 [0.04, 0.04]	0.04 [0.04, 0.04]	0.04 [0.04, 0.04]
Preoperative BNP (pg/mL)	101 [25, 764]	53 [19, 705]	67 [20, 754]

All continuous values are median with interquartile range ([IQR]; RIPC, remote ischemic preconditioning; OR, operating room; BSA, body surface area; ACE-I, angiotensin converting enzyme inhibitor; MRA, mineralocorticoid antagonist; PGE, prostaglandin-E; BNP, B-type natriuretic peptide

**Table 2:** Details in the operating room and of the procedure

	Control	RIPC	Total
	(n = 39)	(N = 45)	(n = 84)
RACHS-1 category	3 [2, 4]	3 [2, 4]	3 [2, 4]
2	11 (28%)	15 (33%)	26 (31%)
3	14 (36%)	13 (29%)	27 (32%)
4	6 (15%)	10 (22%)	16 (19%)
6	8 (21%)	7 (16%)	15 (18%)
Total blood volume prime (mL/kg)	565 [557, 793]	565 [557, 767]	565 [557, 781]
Cardioplegia (mL/kg)	250 [166, 390]	231 [145, 512]	250 [163, 414]
Number of bypass runs			
1	30 (77%)	35 (80%)	65 (78%)
2	9 (23%)	9 (20%)	18 (22%)
Total cardiopulmonary bypass time (minutes)	118 [82, 148]	107 [70, 133]	114 [76, 143]
Circulatory arrest time (minutes)	0 [0, 2]	0 [0,2]	0 [0,2]
Aortic X-clamp time (minutes)	74 [45, 106]	74 [42, 96]	73 [46, 103]
Ischemia time (minutes)	41 [0, 74]	35 [2, 68]	36 [4, 74]
Dexamethasone administered	23 (59%)	29 (64%)	52 (62%)
Number of cycles of cuff inflation	4 [4,4]	4 [4,4]	4 [4,4]

All continuous values are median with interquartile range [IQR]. RACHS, risk adjustment for congenital heart surgery; RIPC, remote ischemic preconditioning

The effect of RIPC on markers of kidney function was assessed by the change in serum creatinine and cystatin C (Table 3). There was no difference between the control and RIPC group in their creatinine or cystatin C at each time point from baseline to 72 h. There is a trend toward a larger decrease in creatinine and cystatin C in the RIPC group denoted by the difference in the slope of the concentration of each biomarker, with a statistical significance in cystatin C (*p* = 0.042), although the magnitude is small (Table 3). The eGFR and its change using formulas based on creatinine and cystatin C also showed a greater increase in the RIPC group (NS).

**Table 3:** Effect of remote ischemic preconditioning on kidney function markers over time

	N	Control	RPC	Difference	*p* value
		Mean (95% CI)	Mean (95% CI)	(95% CI)	
Creatinine (mg/dL)					
Baseline	84	0.39 (0.33, 0.45)	0.38 (0.33, 0.44)	− 0.006 (− 0.01, 0.080)	0.898
6 h	84	0.41 (0.35, 0.47)	0.41 (0.35, 0.47)	0.002 (− 0.083, 0.087)	0.967
12 h	83	0.44 (0.38, 0.50)	0.46 (0.40, 0.51)	0.015 (− 0.071, 0.100)	0.735
24 h	78	0.50 (0.44, 0.56)	0.45 (0.39, 0.50)	− 0.056 (− 0.142, 0.031)	0.188
48 h	71	0.40 (0.34, 0.47)	0.38 (0.32, 0.44)	− 0.021 (− 0.109, 0.067)	0.638
72 h	65	0.38 (0.32, 0.45)	0.33 (0.27, 0.39)	− 0.052 (− 0.140, 0.036)	0.247
Difference in slopes (95% CI)^#^		− 0.0007 (− 0.0015, 0.0002)			**0.109**
Cystatin C (mg/L)					
Baseline	84	1.23 (1.10, 1.35)	1.25 (1.13, 1.37)	0.02 (− 0.15, 0.19)	0.822
6 h	84	0.95 (0.83, 1.08)	1.01 (0.89, 1.12)	0.05 (− 0.12, 0.22)	0.547
12 h	84	1.01 (0.88, 1.13)	1.06 (0.95, 1.18)	0.06 (− 0.12, 0.23)	0.530
24 h	78	1.14 (1.01, 1.27)	1.16 (1.04, 1.27)	0.02 (− 0.16, 0.19)	0.862
48 h	70	1.10 (0.97, 1.23)	1.09 (0.97, 1.21)	− 0.005 (− 0.18, 0.17)	0.959
72 h	65	1.16 (1.03, 1.29)	1.06 (0.94, 1.19)	− 0.10 (− 0.27, 0.08)	0.300
Difference in slopes (95% CI)^#^		− 0.002 (− 0.003, − 0.0001)			**0.042**
eGFR-Cr* (mL/min/1.73 m^2^)					
Baseline	84	108 (89, 126)	102 (85, 120)	− 5 (− 31, 20)	0.686
6 h	84	89 (70, 107)	89 (72, 106)	0.2 (− 25, 26)	0.986
12 h	83	91 (73, 110)	76 (58, 93)	− 15 (− 41, 10)	0.234
24 h	78	94 (75, 113)	87 (70, 105)	− 7 (− 33, 19)	0.612
48 h	71	117 (98, 137)	104 (86, 122)	− 14 (− 41, 13)	0.300
72 h	65	99 (79, 119)	112 (93, 131)	13 (− 14, 41)	0.340
Difference in slopes (95% CI)^#^		0.16 (− 0.16, 0.48)			**0.323**
eGFR-CysC** (mL/min/1.73 m^2^)					
Baseline	84	65 (57, 73)	66 (58, 74)	0.9 (− 10, 12)	0.873
6 h	84	80 (72, 88)	80 (72, 88)	0.2 (− 11, 11)	0.977
12 h	84	79 (70, 87)	77 (69, 84)	− 2 (− 13, 9)	0.745
24 h	78	70 (62, 78)	73 (65, 80)	3 (− 8, 14)	0.628
48 h	70	72 (63, 80)	73 (65, 80)	3 (− 10, 13)	0.840
72 h	65	68 (60, 77)	74 (66, 82)	5 (− 6, 17)	0.360
Difference in slopes (95% CI)^#^		0.07 (− 0.03, 0.16)			**0.157**

Bold value indicates the important value under each subgroupp of the raw data

*eGFR-Cr = 0.413 × (height/serum creatinine)

**eGFR-CysC = 70.69 × (cystatin-C)^−0.931^

^#^The unit for the slope of cystatin C and creatinine is mg/L/h

AKI was also assessed. There were 21 (54%) of patients who met the criteria for AKI in the control group versus 16 (36%) in the RIPC group with an odds ratio 0.31 (*p* = 0.094, 95% CI 0.20–1.14). When adjusted for the baseline creatinine, the odds ratio for the development of AKI in the RIPC group was 0.31 (*p* = 0.037, 95% CI 0.11–0.93). When further adjusted for age, sex, and race, the odds ratio for the development of AKI in the RIPC group was 0.34 (*p* = 0.056, 95% CI 0.11–1.03).

We examined the effect of RIPC on myocardial reperfusion injury, represented by serum troponin I and serum BNP, over the same time course as creatinine and cystatin C (Additional file 2: Table S2). In the treatment group, troponin I was lower than the control group at 6 h, the time point where the peak concentration was observed (*p* = 0.140). Over time, the slope of the concentration of troponin I was not different between the two groups. We also examined BNP and there was no statistically significant difference at each time point or change over time.

Post-operative clinical end points were examined and shown in Table 4. These included length of stay, near-term all-cause mortality, systolic function by echocardiogram, and a composite outcome of dialysis, need for transplant, or mortality. There were no significant differences in any of these conventional post-cardiac surgical outcome measures between study groups. There were 3 composite endpoints met in the entire cohort, all in the RIPC group and its comparison to the control group was not statistically significant. One patient was listed for transplant and survived, and 2 patients died one of which was also on CRRT.

**Table 4:** Association of remote ischemic preconditioning and clinical end points

	Control	RPC	*p* value	Total
	(n = 39)	(N = 45)		(n = 84)
CICU length of stay (days)	3 [1, 8]	4 [2, 9]	0.662	4 [1, 8]
Hospital length of stay (days)	8 [5, 15]	11 [5, 22]	0.267	10 [5, 17]
In-hospital or 30-day mortality	0 (0%)	2 (4.4%)	0.183	2 (2.4%)
Preop ventricular function				
Normal	37 (95%)	42 (93%)	0.640	79 (94%)
Mild dysfunction	2 (5%)	2 (4%)		4 (5%)
Moderate to severe	0 (0%)	1 (2)		1 (1%)
Immediate post-op ventricular function in OR				
Normal	30 (77%)	30 (67%)	0.297	60 (71%)
Mild dysfunction	7 (18%)	8 (18%)		15 (18%)
Moderate to severe	2 (5%)	7 (16%)		9 (11%)
Subsequent post-op ventricular function				
Normal	31 (80%)	32 (71%)	0.676	63 (75%)
Mild dysfunction	5 (13%)	8 (18%)		13 (16%)
Moderate to severe	3 (8%)	5 (11%)		8 (10%)
Time interval of echocardiogram from preop to procedure in OR (days)	15 [10, 67]	18 [10, 74]	0.382	15 [9, 68]
Time interval of echocardiogram in the OR to discharge or closest follow-up during hospitsalization (days)	7 [4, 8]	6 [4, 8]	0.522	6 [4, 8]
Composite of dialysis, listed for heart or kidney transplant, 30-day mortality, in-hospital mortality				
No	39 (100%)	42 (93%)	0.101	81 (96%)
Yes	0 (0%)	3 (7%)		3 (4%)

No patients were withdrawn from the study after undergoing intervention. There were no safety concerns, adverse events, complications, or unintended effects related to the study or its intervention.

## Discussion

In SCRIPT, we studied the clinical and biomarker effects of RIPC in children undergoing moderate to high complexity congenital heart repair. Given the constraints in sample size in clinical trials in this patient population, we focused on renal and cardiac specific biomarkers, surrogates associated with clinical outcomes. Development of AKI early post-operatively [1, 2, 14] or a rise in serum creatinine [15] are associated with major adverse events. Troponin is highly specific to myocardial tissue and a marker of myocardial injury. Its elevation, especially within 24 h of cardiac surgery, is associated with clinical events [16, 17]. The study did not meet the primary end points of an improvement in serum creatinine or troponin I. We observed a small magnitude of improvement in the change of cystatin C compared to baseline in the RIPC group, and less patients developing AKI over the first 72 h. There was no suggestion of a protective effect to myocardial injury in the examination of troponin I and BNP during this same interval. There was also no difference between the groups in cardiac or kidney clinical events during the hospitalization.

We included kidney protection as a primary aim because RIPC delivers a systemic, multiorgan effect beyond the organ that undergoes the major ischemic-reperfusion injury [4]. Furthermore, multiple studies report the high prevalence as well as the clinical implication of AKI post-cardiac surgery [15, 18]. Other studies have included kidney biomarkers, but the results are mixed. McCrindle et al. did not observe a difference in BUN, Cr, cystatin C and AKI in their RIPC group; there was also no difference in clinical outcomes [8]. In other pediatric experiences, Pederson et al. did not demonstrate a difference in Cr, eGFR, cystatin C, NGAL, urine output or need for renal replacement therapy in the RIPC group in a study that involved mostly patients in the RACH-1 2–3 categories [19]. However, Kang et al. studied 249 RIPC versus 200 control subjects in a randomized trial and demonstrated more AKI in the control group [20]. The difference in this study is that RIPC was delivered 12 h before surgery. It is unclear if the earlier timing makes a difference as there are other factors amongst the different studies. An earlier delivery of RIPC is also not an accepted method in adult cardiac studies. In the larger literature of adults undergoing cardiac surgery, for those that included kidney function, the results are mixed [4, 21, 22].

Other differences could be the use of anesthetics. The study by McCrindle et al. used propofol which is considered to have an inhibitory effect on RIPC. In our study, induction of anesthesia utilized a volatile approach (Sevoflurane) with limited use of intravenous anesthetic followed by volatile agents (Isoflurane) and high dose opiate in the maintenance of anesthesia. But numerous anesthetics and pre-medications can be administered in the course of a procedure and it is difficult to control for them in the management of patients in the operating room.

The literature on cardio-protection from RIPC, typically represented by troponin and major cardiovascular events, is also mixed. The results are less positive in the pediatric than adult cardiac surgery studies. Cheung et al. showed an improvement in troponin, inotropic requirement, and airway resistance in the RIPC group; kidney function was not assessed [5]. Wu et al. did not observe a difference in intensive care related clinical end points [7]. McCrindle et al*.* also observed no difference in troponin, length of stay [8]. Zhou et al. observed a lower creatine kinase MB at all time points but only a lower troponin at 2 and 4 h but not 12 and 24 h [6]. Jones et al. examined only neonates with transposition of the great arteries and hypoplastic left heart syndrome and did not observe a difference in troponin [9]. In the cardiovascular studies of adults not all involved cardiac surgery with cardiopulmonary bypass; many investigated the ability of RIPC to lessen injury from acute coronary syndrome when managed by percutaneous revascularization intervention. Among the studies that investigated cardiac surgery, many are related to coronary revascularization alone or revascularization with concomitant valve repair. Hence, the comorbidities, indication for cardiac intervention, type of intervention, and the native stress response of patients in these studies are very different from the pediatric congenital heart disease surgical population. The issue of the extent of exposure to inhaled anesthetics will likely be different as well. The ability to discern hard clinical end points and major adverse cardiovascular events are probably more likely in these adult populations. For example, the number of major renal or cardiovascular adverse events was exceedingly low in the current study population (N = 3) despite the moderate-high complexity nature of the procedures undertaken. In one of the largest registry studies (N = 2240) on AKI after cardiac surgery in neonates, although AKI was common after surgery (53.8%), only extremely severe, stage 3 cardiac surgery-associated acute kidney injury (> 3.0X baseline creatinine or receipt of dialysis with oliguria, present in 9.1%), was independently associated with hospital mortality. This underscores the difficulty in developing an intervention that can actually deliver clinical impact for this patient population (18).

### Limitations

The low event rate along with a modest sample size that is heterogeneous in its clinical characteristics probably limited the ability of the study to detect small differences in renal and cardiac protection between groups. This potential type II error is difficult to overcome in pediatric cardiovascular diseases without a large multicenter trial. The available cardiac biomarkers may not be accurate nor sensitive enough to discriminate cardiac injury in a heterogeneous population. Their kinetics can differ by age and by the type of injurious and protective exposure unrelated to RIPC. The accepted standard to estimate kidney injury and function by serum creatinine, calculated eGFR, and AKI confront similar issues in this congenital heart disease population. The equations used to estimate GFR using creatinine and cystatin c are derived from children with chronic kidney disease, and many of our subjects were of a young age, including less than 12 months of age, where their kidney function may still be evolving. Lastly, since the analysis of the data, newer equations have come out for eGFR for those above 12 months of age, but they have not been used in practice in the cardiac population at our institution. Irrefutable clinical end points such as acute renal failure requiring dialysis, cardiac failure requiring mechanical circulatory support, or in-hospital mortality or organ replacement would require a much larger sample size and a higher risk population. Although an intention to treat analysis is conceptually correct, in this unique study environment, we had to analyze only those who went to the operating room and had the procedure performed as planned. It is unclear if these 16 patients would have altered the results.

## Conclusion

In SCRIPT, RIPC at the time of moderate-high complexity cardiac surgery with cardiopulmonary bypass in children resulted in a general trend toward improved kidney function based on the proportion of patients who developed AKI and the change in cystatin C. However, the magnitude is small and would not be of any clinical impact. There were no indications of a cardioprotective effect. The overall clinical benefits were not apparent. Given the mixed results with RIPC in the adult and pediatric population, and the overall low adverse clinical event rate from cardiac surgery, future investigations on the benefits of RIPC should be focused on even higher risk patients. Such higher risk patient groups include those: with pre-existing kidney or cardiac dysfunction; who require high complexity or riskier surgical procedures; at the time of heart transplant where allograft and kidney dysfunction early post-operatively are common and lead to poor clinical outcome. In furthering the ability to observe a difference with RIPC, a predictive or prognostic enrichment approach can be applied. This can be done by utilizing covariates, biologic or clinical, that are associated with poor kidney outcomes as inclusion criteria. Machine learning using registry data may be able to contribute to the identification of high risk subphenotypes in kidney dysfunction.

### Supplementary Information

**Additional file 1. Supplemental Table 1.** Eligible procedures categorized by Risk Adjustment for Congenital Heart Surgery-1(RACHS-1) risk category: 1–6.

**Additional file 2. Supplemental Table 2.** Effect of ischemic preconditioning on cardiac biomarkers over time.

## Data Availability

The dataset can be accessed with the permission of the authors.
