# Supplementary material for: Randomized controlled trial of remote ischemic preconditioning in children having cardiac surgery
Source: J Cardiothorac Surg. 2024 Jan 3;19:5. doi: 10.1186/s13019-023-02450-8 (PMC10765905; doi:10.1186/s13019-023-02450-8)
Supplement: Supplementary file 1 — Additional file 1. Supplemental Table 1. Eligible procedures categorized by Risk Adjustment for Congenital Heart Surgery-1(RACHS-1) risk category: 1–6. [file 13019_2023_2450_MOESM1_ESM.docx]

Supplemental Table 1. Eligible procedures categorized by RACHS-1 risk category:

**Risk category 1**

None

**Risk category 2**

- Aortic valvotomy or valvuloplasty at age >30 d
- Subaortic stenosis resection
- Pulmonary valve replacement
- Right ventricular infundibulectomy
- Pulmonary outflow tract augmentation
- Atrial septal defect and ventricular septal defect repair
- Atrial septal defect primum repair
- Ventricular septal defect repair +/- PA band removal
- Ventricular septal defect closure and pulmonary valvotomy or

infundibular resection

- Total repair of tetralogy of Fallot
- Repair of total anomalous pulmonary veins at age >30 d

**Risk category 3**

- Aortic valve replacement
- Ross procedure
- Ventriculomyotomy
- Mitral valvotomy or valvuloplasty
- Mitral valve replacement
- Tricuspid valvotomy or valvuloplasty
- Tricuspid valve replacement
- Tricuspid valve repositioning for Ebstein anomaly at age >30 d
- Repair of double-outlet right ventricle with or without repair of

right ventricular obstruction

- Repair of transitional or complete atrioventricular canal with or

without valve replacement

- Repair of tetralogy of Fallot with pulmonary atresia
- Arterial switch operation
- Repair of coarctation and ventricular septal defect closure
- Right ventricular to pulmonary artery conduit
- Left ventricular to pulmonary artery conduit

**Risk category 4**

- Aortic valvotomy or valvuloplasty at age ≤30 d
- Konno procedure
- Repair of total anomalous pulmonary veins at age ≤30 d
- Repair of transposition, ventricular septal defect, and sub-pulmonary

stenosis (Rastelli)

- Repair of truncus arteriosus
- Repair of hypoplastic or interrupted arch without ventricular septal

defect closure

- Repair of hypoplastic or interrupted aortic arch with ventricular

septal defect closure

- Transverse arch graft (augmentation)
- Arterial switch with VSD closure +/- PA band removal
- Arterial switch with repair of sub-pulmonary stenosis
- Repair of truncus arteriosus

**Risk category 5**

- Repair of truncus arteriosus and interrupted arch

**Risk category 6**

- Stage 1 repair of hypoplastic left heart syndrome (Norwood operation)
- Stage 1 repair of non-hypoplastic left heart syndrome conditions
- Damus-Kaye-Stansel procedure

Randomized Controlled Trial of Remote Ischemic Preconditioning in Children Having Cardiac Surgery. Pediatric Cardiology. Yuk Law, corresponding author. Seattle Children’s Hospital. [Yuk.law@seattlechildrens.org](mailto:Yuk.law@seattlechildrens.org).
