# Supplementary material for: Randomized controlled trial of remote ischemic preconditioning in children having cardiac surgery
Source: J Cardiothorac Surg. 2024 Jan 3;19:5. doi: 10.1186/s13019-023-02450-8 (PMC10765905; doi:10.1186/s13019-023-02450-8)
Supplement: Supplementary file 2 — Additional file 2. Supplemental Table 2. Effect of ischemic preconditioning on cardiac biomarkers over time. [file 13019_2023_2450_MOESM2_ESM.docx]

|  | **N** | **Control** | **RPC** | **Difference** | **p-value** |
| --- | --- | --- | --- | --- | --- |
|  |  | (95% CI) | (95% CI) | (95% CI) |  |
| **BNP (pg/mL)** |  |  |  |  |  |
| baseline | 79 | 474 (251, 697) | 535 (327, 743) | 61 (-243, 366) | 0.694 |
| 6 hours | 37 | 462 (193, 731) | 498 (240, 755) | 36 (-336, 408) | 0.851 |
| 12 hours | 46 | 413 (153, 674) | 679 (444, 914) | 266 (-85, 617) | 0.137 |
| 24 hours | 64 | 489 (253, 725) | 603 (385, 820) | 114 (-207, 435) | 0.487 |
| 48 hours | 64 | 493 (255, 731) | 620 (404, 836) | 126 (-195, 448) | 0.441 |
| 72 hours | 61 | 408 (172, 644) | 520 (298, 742) | 112 (-212, 436) | 0.499 |
| Difference in slopes (95% CI) |  | 0.43 (-2.97, 3.82) | |  | 0.806 |
| **Troponin I (ng/mL)** |  |  |  |  |  |
| baseline | 84 | 0.04 (-1.24, 1.33) | 0.04 (-1.16, 1.24) | -0.004 (-1.76, 1.76) | 0.997 |
| 6 hours | 80 | 6.68 (5.38, 7.99) | 5.34 (4.13, 6.56) | -1.34 (-3.12, 1.76) | 0.140 |
| 12 hours | 76 | 4.08 (2.77, 5.39) | 3.68 (2.45, 4.91) | -0.40 (-2.20, 1.40) | 0.662 |
| 24 hours | 75 | 3.72 (2.40, 5.04) | 2.84 (1.61, 4.07) | -0.88 (-2.69, 0.92) | 0.338 |
| 48 hours | 71 | 2.35 (1.01, 3.69) | 2.12 (0.87, 3.37) | -0.23 (-2.06, 1.60) | 0.807 |
| 72 hours | 65 | 1.03 (-0.34, 2.39) | 1.57 (0.29, 2.86) | 0.55 (-1.33, 2.42) | 0.568 |
| Difference in slopes (95% CI) |  | 0.01 (-0.01, 0.04) | |  | 0.283 |

Supplemental Table 2. Effect of ischemic preconditioning on cardiac biomarkers over time.

Randomized Controlled Trial of Remote Ischemic Preconditioning in Children Having Cardiac Surgery. Pediatric Cardiology. Yuk Law, corresponding author. Seattle Children’s Hospital. [Yuk.law@seattlechildrens.org](mailto:Yuk.law@seattlechildrens.org).
